# Colonic metastasis of renal cell carcinoma following curative nephrectomy: A case report and review of the literature

**DOI:** 10.1016/j.ijscr.2019.10.035

**Published:** 2019-10-23

**Authors:** Özkan Subaşı, Mehmet Aziret, Kerem Karaman, Metin Ercan

**Affiliations:** Sakarya University Education and Research Hospital, Department of General Surgery, Sakarya, Turkey

**Keywords:** Colon, Metastasis, Renal cell carcinoma

## Abstract

**Introduction:**

Renal cell carcinoma (RCC) is a rare tumor that comprises only 3% of adult cancers, while renal parenchymal tumors constitute 85% of all RCC cases. RCC frequently metastasizes to the lungs, bones, brain or liver; however, the gastrointestinal tract, particularly the colon, is an unusual location for metastasis.

**Case report:**

A 63-year-old male patient was admitted complaining of hematochezia. The patient had undergone left-side nephrectomy for RCC, 5 years previously. Computed tomography and colonoscopy detected a splenic flexure tumor and after left hemicolectomy and splenectomy, histopathological examination revealed a colonic metastasis of the renal cell carcinoma.

**Discussion:**

Cases of colonic metastasis following resection of a RCC are uncommon in the literature and their location can be very varied, but include the sigmoid colon, splenic flexure, transvers colon and hepatic flexure. Recurrence of RCC is frequently seen during the first three postoperative years, and surgical resection is suggested for solitary non-metastatic tumor.

**Conclusion:**

RCC rarely metastases to the colon but may occur years after curative resection. Therefore, RCC patients should be closely followed for the long term. In case of isolated metastasis, long-term survival can be achieved with R0 resection.

## Highlights

• Colonic metastasis of the renal cell carcinoma (RCC) following curative nephrectomy is an extremely rare.
• Early diagnosis of colonic metastasis of the RCC is an important for survival.
• Surgical resection is a effective treatment method for colonic metastasis of the RCC.

## 1 Introduction

Renal cell cancer (RCC) is a primary tumor of the kidney, and is associated with the highest mortality rate (40%) of all patients with urinary tract tumors [1,2]. Accompanying metastatic disease is very common and diagnosed in 25% of all patients. Moreover, there is no time limit to the metastatic activity with late metastatic disease diagnosed after a 5-year period in 10% of patients. Similarly, metastasis occurs even after curative resection with R0 in approximately 40% of patients [3,4]. Most metastases are located in the lungs (75%), lymph nodes (36%), bone (20%) or liver (18%) [5]. Ultrasound, magnetic resonance imaging, colonoscopy, arteriography and PET-CT (positron emission tomography/computed tomography) are all useful for diagnosis, staging and management of the disease, although contrast enhanced - thin-slice CT has a higher sensitivity for evaluating local recurrence and metastatic disease [3,5,6]. The gastrointestinal tract is an unusual location for metastases, and less than 15 patients are recorded in the literature as undergoing curative nephrectomy for late period metastatic RCC [[6], [7], [8], [9], [10], [11], [12], [13], [14], [15]].

In this case report, we present a patient who was managed successfully with colon resection for late colonic metastasis of RCC.

## 2 Material and methods

### 2.1 Study protocol and design

**Fig. 1:**
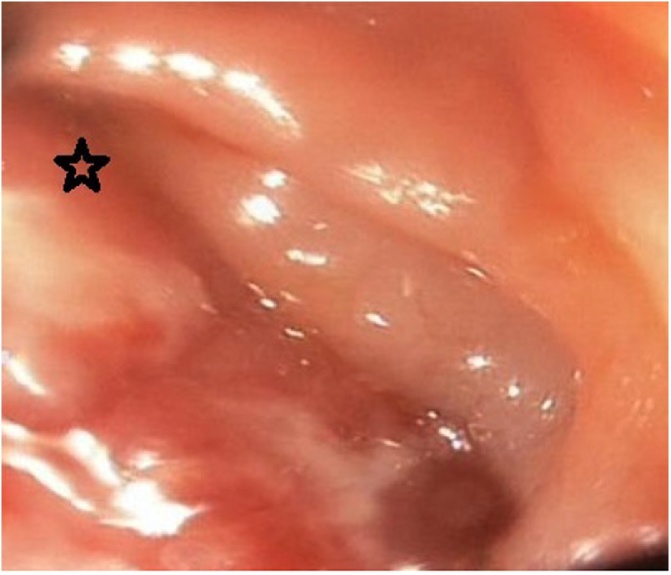
Colonoscopy reveals a partially obstructive mass in the left colon (Arrow).

Research identification and data extraction were realised by searching PubMed, Google Scholar, Research gate, Scopus, Ovid and Cochrane Database of Systematic Reviews using the following search terms: ‘renal cell cancer’, ‘metastasis’, ‘colon’ plus ‘recurrence’. In addition, all relevant references were manually investigated by trained researchers to find additional studies. Titles, abstracts, key words and full-texts of the articles were assessed for inclusion and exclusion criteria. Full-texts were used wherever possible for more accurate evaluation. Our literature review included all articles from 1991 to April 2019 from which we harvested the following information: first author of article and year of publication, age of patient, recurrence year, symptoms, metastatic location, treatment method, details of surgery. Finally, we formed a search flow diagram according to the data evaluation (Fig. 1).

## 3 Results

### 3.1 Case study

A 63-year-old male patient with a history of left-side nephrectomy for RCC was admitted for abdominal pain, nausea and hematochezia. In the 5-year postoperative follow up, a 5 cm tumor was detected in the left colon during colonoscopy. The mass was located in the colon wall with mucosa of smooth appearance (Fig. 1).

**Fig. 2:**
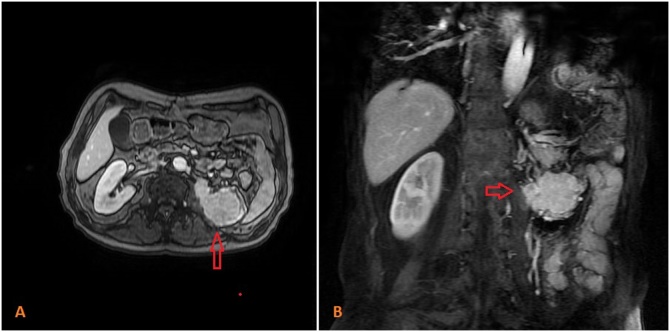
A colonic tumor in abdominal MRI (A and B).

**Fig. 3:**
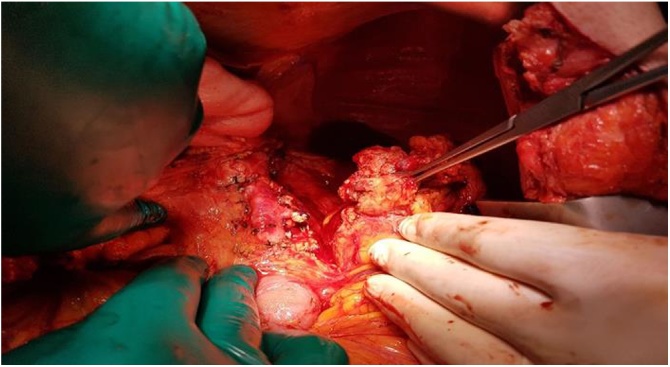
An invasive tumor of the splenic flexure during operation.

**Fig. 4:**
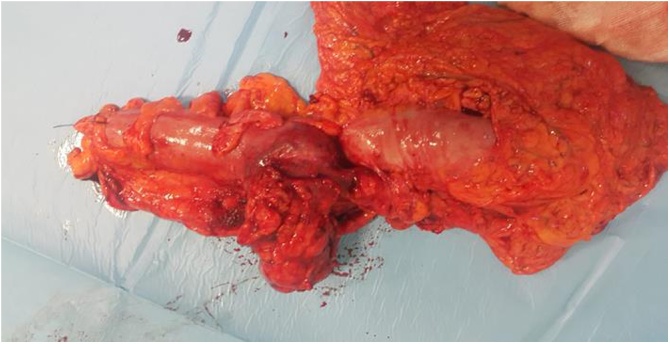
Specimen after resection.

At biopsy, histopathological examination indicated a malign epithelial tumor metastasis. Magnetic resonance imaging (MRI) revealed a tumoral mass near the anterolateral side of the psoas muscle in the left-nephrectomy region (Fig. 2). After oncology consultation, the patient underwent laparotomy, where a hemorrhagic, 5 cm recurrent mass invading both colon and spleen was found at the splenic flexure (Fig. 3, Fig. 4). In addition to the colonic obstruction caused by the tumor, multiple lymph nodes were detected in the para-aortic region during the operation. Left hemicolectomy with Hartmann’s ostomy, splenectomy and para-aortic lymph dissection was performed without complication. On the 8^th^ postoperative day, an abscess was detected in the splenectomy area on CT. The abscess was drained with an external drainage catheter by an interventional radiologist. The patient was discharged on the 12^th^ postoperative day after removing the external drainage catheter. In histopathological examination of the surgical specimen, a metastasis of the clear cell renal carcinoma was confirmed with a size of 4.7 × 3.8 × 3.5 cm and intact surgical margins. The tumor was extending from the serosal layer to the submucosa in the colon. The number of metastatic lymph node was one in total of fourteen lymph nodes in para-aortic region. Moreover, the spleen was not involved by tumor. The patient has been followed up by oncology and is currently asymptomatic in the postoperative 6^th^ month.

### 3.2 Literature review

Examination of English language medical databases, using Google Scholar, Research-gate, Scopus and PubMed, revealed a total of 12 cases of renal cell carcinoma following curative nephrectomy between 1991 and 2019. Most patients were male (83%; 10 patients), and the median age was 64 years (min-max: 35–84). The median recurrence period was 7 years (min-max: 2–17). The majority of the patients presented with hematochezia (41.6%; 5 patients) and abdominal pain (41.6%; 5 patients). Furthermore, metastases were most frequently detected in the splenic flexure (33.3%; 4 patients) and transvers colon (16.6%; 2 patients). According to the literature review, left hemicolectomy ± splenectomy was performed in 4 patients (33.3%), right hemicolectomy in 4 patients (33.3%), transverse colectomy in 2 patients (16.6%) and anterior resection in 2 patients (16.6%). This study has been presented in line with SCARE criteria [16].

## 4 Discussion

RCC is a primary tumor of the kidney, which is most frequently seen in adulthood, usually in the sixth and seventh decades and most commonly in men (M/F: 2/1) [17]. Although most RCCs are sporadic, 4% of these tumors are familial, and they are associated with certain syndromes such as Von Hippel-Lindau disease, tuberous sclerosis, hereditary papillary renal cancer, Birt-Hogg-Dube syndrome, hereditary leiomyoma, familial renal oncocytoma and hereditary renal cancers [18].

The recurrence rate after curative surgery of RCC patients is 20–40% [8]. Although recurrences tend to occur within the first 5 years after primary surgery, approximately 5–10% have late recurrences after the first 5 years [18,19]. Uchida et al. reported recurrence in 68 of 239 (28.4%) non-metastatic patients [19]; with recurrence within the first five years in 84% cases. In addition, McNichols et al. reported a late metastasis rate of 11% in long-term survival patients (over ten years) following nephrectomy [20]. In our case, recurrence was seen 5 years after curative nephrectomy.

RCC has the potential to metastasize to various sites and location of metastases may vary according to the late or early time of recurrence [20]. Metastatic spread may be by lymphatic, hematogenous or direct invasion. Lymph node and distant metastases are seen even in early stage RCCs and the risk of metastasis increases with tumor size [21]. Although RCC colonic metastasis is very rare, it can metastasize to the whole gastrointestinal tract and there is no specific lymphatic or hematogenous pathway that can effectively explain colonic metastasis [5,21]. The sites of colonic metastasis also vary; although most commonly, the sigmoid, splenic flexure, transverse colon and hepatic flexure are involved. Furthermore, the prognosis for non-surgically treated disease in metastatic patients is poor [21,22].

Because of the higher metastasis rate, management of the RCC requires a multidisciplinary approach. Both the National Comprehensive Cancer Network (NCCN) and American Urology Association (AUA) suggest routine postoperative surveillance for the first 5 years. However, although there is no clear recommendation for a longer follow up period, in their assessment of 3651 operated patients, Stewart et al. showed a reduction in recurrences when patients were followed up for a longer period [23]. Therefore, because of the potential late RCC recurrence, postoperative surveillance may need to be extended beyond 5 years.

In the literature, surgical treatment is suggested for both solitary and oligo-metastatic disease [22,23]. In patients with solitary metastatic disease, surgical resection has a high disease-free and long-term survival rate [4,22,23]. In addition, in a study by Kavolius et al. of 141 metastatic patients who underwent surgery, patients with negative surgical margins had a higher disease-free survival rate than patients with non-curative or non-surgical treatment [23]. Kavolius et al. suggested favorable features for survival included disease-free intervals greater than 12 months, solitary lesions and age younger than 60 years. They also suggested metastasectomy because of its long-term survival rates of 46% and 44% respectively, following curative resection of second and third metastases [23].

A review of previous literature in Table 1, indicates that patients with colonic metastases were mostly male (83%), and the median age and recurrence year were 64 years (min-max: 35–84) and 7 years (min-max: 2–17), respectively. Patients presented with symptoms of Hematochezia (41.6%) and abdominal pain (41.6%). The metastasis locations were the splenic flexure (33.3%), transvers colon (16.6%), recto-sigmoid (16.6%) and hepatic flexure (8.3%). After diagnosis of the disease, the choice of surgical approach was left hemicolectomy ± splenectomy (33.3%), right hemicolectomy (33.3%), transvers colectomy (16.6%) and anterior resection (16.6%) (Table 2).

**Table 1:** Renal cell carcinoma patients with late metastases.

	Year	Age	Gender	Recurrence (Year)	Location	Symptom	Operation
Ruiz et al. [6]	1991	NA	NA	11	NA	NA	NA
Thomason et al. [7]	1991	71	M	17	Splenic flexure	Hematochezia	Left hemicolectomy
Tokonabe et al. [8]	1996	83	M	7	Transvers colon	Melena	Transvers colectomy
Avital et al. [9]	1998	72	F	5	Right colon	AP, anemia	Right hemicolectomy
Valdespino-Castillo et al. [10]	2008	60	M	8	Splenic flexure	Hematochezia	Left hemicolectomy
Yetkin et al. [11]	2008	60	M	5	Hepatic flexure	Anemia, AP	Right hemicolectomy
Jadav et al. [12]	2010	65	F	9	Transvers colon	AP	Transvers colectomy
Milovic et al. [13]	2013	63	M	2	Sigmoid colon	AP	Left hemicolectomy
		35	M	2	Splenic flexure	Anemia, AP	Right hemicolectomy
		39	M	4	Ileocecal valve	Constipation	Right hemicolectomy
Vo et al. [14]	2016	67	M	9	Recto-sigmoid	Hematochezia	Anterior resection
Zang et al. [15]	2019	84	M	13	Recto-sigmoid	Hematochezia	Anterior resection
**Present study**	**2019**	**63**	**M**	**5**	**Splenic flexure**	**Hematochezia**	**Left hemicolectomy, splenectomy**

AP: Abdominal pain, NA: Not available.

**Table 2:** Patient’s characteristics and treatment.

**Age**	64 (35–84)
**Gender**	M / F: 10 / 2 (83% vs 17%)
**Recurrence year**	7 (2–17)
**Metastatic location**	**n (%)**
Splenic flexure	4 (33)
Transverse colon	2 (16.6)
Recto-sigmoid	2 (16.6)
Hepatic flexure	1 (8.3)
Ileocecal valve	1 (8.3)
Sigmoid colon	1 (8.3)
Right colon	1 (8.3)
**Treatment**	**n (%)**
Left hemi-colectomy ± splenectomy	4 (33)
Right hemi-colectomy	4 (33)
Transvers colectomy	2 (16.6)
Anterior resection	2 (16.6)

F: Female, M: Male, vs: versus.

## 5 Conclusion

Recurrent metastases can develop even many years after curative nephrectomy in RCC. Therefore, long-term close follow-up may be beneficial in patients with a history of curative nephrectomy for RCC. In these patients, potential recurrence or metastasis should always be considered in the case of any abdominal pain, anemia or gastrointestinal bleeding. In addition, R0 resection may provide a survival advantage in patients with colonic metastasis.

## Sources of funding

No funding.

## Ethical approval

This is a case report so the study is exempt from ethical approval in our institution.

## Consent

The patient’s consent was obtained.

## Author’s contribution

Mehmet Aziret – Write and data collection.

Özkan Subaşı – Design and write.

Kerem Karaman – Data analysis.

Metin Ercan – Interpretation.

## Registration of research studies

N/A.

## Guarantor

Mehmet Aziret.

## Provenance and peer review

Commissioned, externally peer-reviewed.

## Declaration of Competing Interest

There is not any conflict of interest.
